# Voices in practice: Exploring genetic counseling ethical, cultural, social, and religious dynamics in the UAE

**DOI:** 10.1002/jgc4.70139

**Published:** 2025-11-14

**Authors:** Hind J. Almarri, Sameera Koodakkadavath, Azhar T. Rahma, Muna Al Saffar

**Affiliations:** ^1^ Department of Genetics and Genomics, College of Medicine & Health Sciences United Arab Emirates University AlAin UAE; ^2^ Institute of Public Health, College of Medicine & Health Sciences United Arab Emirates University AlAin UAE

**Keywords:** cultural competence, ethical challenges, genetic counseling, patient‐centered care, qualitative research, religious beliefs, semi‐directive counseling, United Arab Emirates

## Abstract

Genetic counseling is expanding globally, yet remains underexplored in Middle Eastern contexts. In the United Arab Emirates (UAE), rapid biomedical advancements intersect with traditional sociocultural and religious norms, presenting unique contexts for clinical practice. This study explored the perspectives of genetic counselors and clinical geneticists to identify key sociocultural, ethical, and systemic factors influencing genetic counseling in the UAE. Guided by a constructivist–interpretivist paradigm, we conducted semi‐structured interviews, generating a dataset from 11 professionals (seven genetic counselors, four clinical geneticists) practicing in the UAE between January and August 2024. Data were analyzed using Braun and Clarke's reflexive thematic analysis, reported in accordance with RTARG guidelines. The analysis was predominantly inductive, while the Consolidated Framework for Implementation Research (CFIR) was used deductively as a sensitizing framework for themes relating to institutional and systemic influences. Four major themes were constructed: (1) Social and cultural dynamics, including stigma, limited genetic literacy, and family‐centered decision‐making, influenced engagement and consent; (2) Religious perspectives: faith offered resilience but at times fostered fatalism that limited intervention; (3) Ethical considerations: autonomy, confidentiality, and informed consent were negotiated within collectivist family structures; and (4) Systemic factors, including limited interprofessional coordination, the need for UAE‐specific training and time constraints. The Emirati Genome Program was described as a facilitator of awareness and management. Participants emphasized the need for culturally responsive, semi‐directive counseling approaches, enhanced consent processes, and targeted community education. Our interpretive analysis underscores the need for culturally responsive, semi‐directive counseling approaches that balance respect for autonomy with relational guidance. These insights provide a framework for strengthening practice, training, and policy in the UAE and may be applicable across Gulf and MENA healthcare systems with similar sociocultural dynamics.

**What is known about this topic?**

Genetic counseling practices are shaped by sociocultural and religious contexts, particularly in non‐Western contexts. Yet, empirical research exploring how these dynamics affect counseling delivery and patient decision‐making in the Arab Gulf region remains limited. Existing studies focus primarily on genetic literacy and attitudes toward testing, rather than professional practice or ethical frameworks.

**What this paper adds to the topic?**

This article offers the first reflexive, qualitative exploration of genetic counseling in the UAE. Through participants' narratives, we constructed themes around stigma, religious fatalism, limited genetic literacy, and structural factors as shaping counseling practice. We interpreted counselors as actively navigating these dynamics through culturally sensitive strategies, including a newly conceptualized semi‐directive approach. The findings provide context‐specific contributions to service development, policy, and training, with relevance for other settings where sociocultural and ethical complexities similarly shape practice.

## 1 INTRODUCTION

Throughout the past few decades, advances in molecular technologies, declining costs, and growing testing accuracy and capabilities have accelerated the integration of genetic services into healthcare (Abul‐Husn et al., 2014; Facio et al., 2014), fostering the growth of the genetic counseling profession. Genetic counselors apply genetic and genomic information to support patients and families to make informed and autonomous medical decisions (Genetic Alliance & New York‐Mid‐Atlantic Consortium for Genetic and Newborn Screening Services, 2009). While the significance of this profession is well established in the West (Patch & Middleton, 2018), its practice in the Middle East is shaped by ethical, religious, cultural, and social factors. In the UAE, initiatives such as the launch of the Emirati Genome Program (Department of Health – Abu Dhabi, 2022) reflect a strong institutional commitment to genomic medicine. Yet, access to genetic counseling remains limited, and little is known about the underlying dynamics. The field is formally regulated, with licensure and scope of practice defined by the Department of Health. Services are delivered by clinical geneticists and certified genetic counselors, all of whom, at the time of data collection, had trained abroad. A small number of family physicians also provide standardized pretest counseling as part of the national premarital screening program. In the UAE premarital genetic screening program, upskilled physicians are authorized to provide standardized pretest counseling and to discuss the implications of negative results with couples. Their scope of practice is intentionally limited: in the event of a positive finding, they are permitted to offer only preliminary counseling, with mandatory referral to a clinical geneticist or certified genetic counselor for comprehensive, in‐depth counseling. This framework ensures that couples receive accurate medical and reproductive guidance while maintaining access to appropriate specialist expertise. One significant barrier is social stigma. In the region, many families express concern over social standing and marriage prospects, which may deter disclosure or engagement with services (Al Aqeel, 2007; El Shanti et al., 2015; Jassim et al., 2015; Jumah et al., 2019). Women, in particular, may experience social exclusion as a result of a genetic diagnosis or carrier status (Al‐Shamsi et al., 2023; Clarke, 2016; Elobaid et al., 2014; Karam et al., 2024). Furthermore, in the Middle East, with Islam being the predominant religion, religious beliefs greatly affect attitudes toward genetic counseling usage (Bloomer & Al‐Mutair, 2013; Orzechowski et al., 2021). The Islamic notion of destiny (Qadar), central to Muslims, frames every aspect of existence, such as illness and mortality, as part of a divine plan. Religion was reported to enable engagement with genetic services by reinforcing family‐centered approaches and proactive choices, as shown in British Pakistani communities and clinical experiences in Pakistan (Akbar et al., 2025; Darr et al., 2013; Hanif et al., 2023). Beyond Muslim contexts, research from Western settings has also documented how religion functions as a coping resource in illness and bereavement (Braam et al., 2010; Loewenthal et al., 2001; McIntosh et al., 1993; Thune‐Boyle et al., 2013). While Islamic teachings encourage seeking treatment, misinterpretations of this concept will further complicate decision‐making, sometimes fostering fatalism and discouraging medical intervention such as genetic testing or termination (Ahmed et al., 2006; Al‐Matary & Ali, 2014; Al‐Shahri & Al‐Khenaizan, 2005; Benidir et al., 2023; Schwartz et al., 2000). However, evidence from Saudi Arabia further shows that religious education can mitigate these barriers and increase acceptance of medical options, including termination in severe cases (Alkuraya & Kilani, 2001). Additionally, ethical challenges arise as patients often defer decisions to healthcare providers or family members (Ezenkwele & Roodsari, 2013; Mobeireek et al., 2008). Previous research in the Middle East has highlighted collectivistic traditions, meaning that families are involved in medical decisions, which may conflict with the cornerstones of genetic counseling that emphasize patient autonomy and confidentiality (El‐Hazmi, 2004). Genetic counselors are thus put in a difficult position as they are tasked with balancing patient autonomy with familial and cultural expectations. Adding further complexity, some patients have a preference for a directive approach from healthcare providers (Alfahmi, 2022). Limited genetic literacy across the region, including the UAE, impedes patients' understanding of genetic principles and complicates informed consent (Khdair et al., 2021; Rahma et al., 2023). The social and cultural context strongly shapes the understanding and accessibility of genetic services (Alsulaiman & Hewison, 2006; Balobaid et al., 2016; Bruwer et al., 2014; Malik et al., 2022). For example, consanguinity, a means of maintaining family bonds, further influences perceptions of risk (Iqbal et al., 2022). Although the goal of genetic counseling is to provide informed guidance rather than to change cultural practices (National Society of Genetic Counselors (NSGC), 2018), it can be viewed as a challenge to long‐standing traditions. The UAE has sought to address such challenges by expanding premarital screening and IVF services, framing them as culturally sensitive strategies for reducing genetic risk (Department of Health – Abu Dhabi, 2023). In parallel, international debates in genetic counseling have shifted beyond strict nondirectiveness toward more relational and shared decision‐making models (Biesecker & Peters, 2001; Elwyn et al., 2012; Weil, 2003). However, how these approaches are interpreted and applied in Middle Eastern contexts remains underexplored. Although prior research has investigated genetic literacy, attitudes toward genetic testing, and cultural influences on genetic healthcare, no study has yet comprehensively examined genetic counseling practice in the UAE. This focus is important because the UAE presents a distinct model of rapid modernization that coexists with deeply rooted social, cultural, and religious values. Earlier work in the UAE has drawn attention to the way families interpret genetic information through their own frameworks. For example, Al‐Gazali (2005) documented families' responses to preconception counseling following the diagnosis of a genetic condition in a previous child, noting that some retained alternative and inaccurate explanations despite receiving clear recurrence risk information (Al‐Gazali, 2005). Such work highlights the importance of contextually responsive practices beyond the mere provision of biomedical facts.

This study examines the perspectives of genetic counselors and clinical geneticists in the UAE to illuminate factors shaping practice and to inform strategies for improving accessibility, engagement, and outcomes. As researchers situated within the field of genetic counseling in the UAE, we acknowledge that our positionalities influenced both the questions we asked and our interpretive engagement with participants' accounts. The findings therefore reflect a co‐construction of meaning between participants' experiences and our interpretive lens.

## 2 METHODOLOGY

This study is reported in accordance with the Reporting guidelines for Reflexive Thematic Analysis (RTARG) (Braun & Clarke, 2021, 2024). A qualitative research design was employed, incorporating in‐depth, semi‐structured, virtual interviews with genetic counselors and clinical geneticists practicing in the UAE. The study was positioned within a constructivist–interpretivist paradigm, which assumes that knowledge is co‐constructed through researcher–participant interaction and that multiple contextual realities shape experiences (Wainstein et al., 2023). Participants were recruited purposively to share their perspectives on the provision of genetic counseling services, including the barriers and strategies to improve service delivery. Ethical approval was granted by the IRB Ethics Committee at CMHS‐UAE University, with Reference Number ERSC_2023_3901, dated January 19, 2024. A genetic counseling student, under the supervision of an experienced qualitative researcher and genetic counselor, recruited participants from various healthcare institutions across the UAE. This researcher positioning inevitably shaped recruitment and interaction, and we acknowledge this as part of the co‐construction of the dataset. This study contributes to understanding the landscape of genetic counseling in the UAE, providing insights to inform workforce planning and service improvement.

### 2.1 Study context

At the time of data generation, the genetics workforce in the UAE was highly limited. Nationally, there were nine practicing genetic counselors and eight clinical geneticists serving a population of nearly 11 million, roughly one genetics provider per 650,000 individuals. Two of the clinical geneticists had trained locally in clinical genetics, while all genetic counselors had pursued their training abroad, reflecting the absence of a domestic program in genetic counseling during the study period. Despite this, the profession of genetic counseling was formally regulated, with licensure and a defined scope of practice. A milestone occurred later in 2024 with the launch of the first homegrown master's program in genetic counseling at UAE University, which enrolled its inaugural cohort and marked the beginning of local capacity‐building efforts.

### 2.2 Participant recruitment and response

Participants were recruited purposively to capture a range of perspectives from professionals directly involved in genetic service delivery in the UAE. Inclusion criteria were active practice in the UAE as a genetic counselor, clinical geneticist, or, where feasible, family physician with direct involvement in genetic counseling. Individuals not currently practicing or lacking direct involvement were excluded.

In the UAE, a designated subset of family physicians is authorized to conduct pretest counseling within the national premarital screening program. To obtain this privilege, physicians complete structured training developed by the Department of Health and Emirates Health Services in collaboration with UAE University, followed by formal evaluation. Their role is intentionally limited to providing standardized pretest counseling and discussing negative results. In cases with a family history of genetic conditions, prior genetic testing, or requests for detailed information, physicians must refer to clinical genetics. For positive results, they may only provide preliminary counseling, with all at‐risk couples referred without exception to a clinical geneticist or certified genetic counselor. Importantly, a positive result does not restrict couples from marrying but ensures they receive appropriate counseling and support. Continued authorization requires annual refresher training and evaluation.

No family physicians agreed to participate, which we interpret as likely reflecting workload pressures and limited familiarity with qualitative research. Eleven professionals—seven genetic counselors and four clinical geneticists—consented and completed the interviews (Table 1). All participants who consented were included in the final analysis. None had received UAE‐specific training in genetic counseling, though several identified as Emirati or Arab or reported cultural and linguistic familiarity, which they described as beneficial in practice.

**TABLE 1:** Demographic and professional characteristics of study participants (*n* = 11).

Characteristic	Genetic counselors (*n* = 7)	Clinical geneticists (*n* = 4)	Total (*n* = 11)
Sex	5 Females, 2 Males	4 Females	9 Females, 2 Males
Total professional experience	Range: 5–16 years	Range: 2–20+ years	2–20+ years
Experience in UAE	Range: 3–9 years	Range: 2–19 years	2–19 years
Training location	All abroad	2 local and 2 abroad (clinical genetics)	All GCs abroad; 2 CGs local and 2 abroad (clinical genetics)
UAE‐specific GC Training	None	None	None
Ethnic/cultural background	Predominantly non‐Arab (some Arab)	All Arab	Mixed backgrounds

*Note*: Demographic data are presented in aggregated form to protect participant confidentiality, given the small national workforce. Participants were categorized by sex and professional characteristics for demographic description; no other classifications or demographic details were included in this dataset. Participant codes indicate professional group and sex: “GC” = genetic counselor, “MG” = clinical geneticist, “F” = female, and “M” = male.

## 3 PARTICIPANT CHARACTERISTICS

### 3.1 Data generation

Data were generated over a 7‐month period from January to August 2024. Participants received an information sheet outlining the study's aims, procedures, voluntary nature, and confidentiality measures. Written informed consent was obtained electronically before each interview, along with verbal confirmation at the beginning of the session. Consent included permission for audio recording and use of anonymized quotations. Interviews were conducted virtually via Microsoft Teams and lasted about 60 min each. An interview guide (see File S1) was developed from the literature and reviewed by a senior qualitative researcher and experienced genetic counselor for clarity and conceptual fit. Field notes captured contextual details and interviewer reflections. Audio files were transcribed orthographically, and transcripts were independently reviewed by two researchers for accuracy. The dataset was shaped by information power (Malterud et al., 2016), taking into account the study's focused aim, the participants' direct expertise, the quality of dialogue, and the analytic strategy, rather than by notions of saturation. All data were anonymized and securely stored on password‐protected devices. Audio recordings were deleted following transcription.

### 3.2 Data analysis

Our primary analytic approach was reflexive thematic analysis (RTA), following six phases of Braun and Clarke (2006, 2021, 2024). The analysis was primarily inductive and semantic, with some attention to latent cultural and systemic dimensions. Two researchers engaged in ongoing reflexive dialogue to enrich interpretation; inter‐coder reliability statistics were not calculated, as the aim was interpretive depth and thematic coherence rather than coding consensus (Braun & Clarke, 2021, 2024). An iterative coding strategy was used to develop and refine codes, which were subsequently organized into themes and subthemes using NVivo software, while coding decisions remained interpretive. Themes were refined collaboratively to ensure conceptual clarity and richness of interpretation.

To deepen interpretation of institutional and contextual influences, a subset of the data was examined deductively using the Consolidated Framework for Implementation Research (CFIR) domains (inner setting, outer setting, and individual characteristics), offering a theoretical lens to support a richer understanding of implementation barriers and enablers. This deductive component complemented the inductive thematic analysis and did not function as a coding framework or validation tool. Visualizations were created with Napkin AI and Lucidchart to illustrate thematic patterns and the semi‐directive model.

## 4 POSITIONALITY STATEMENT

This positionality statement is provided to offer readers transparency regarding the perspectives that informed this research. HJA is an Emirati genetic counseling student, trainee, and researcher embedded within the national healthcare system. Her insider perspective facilitated access and contextual understanding while requiring ongoing reflexivity to minimize potential bias. SK is a public health student who brought an external disciplinary lens, supporting reflexive dialogue and alternative perspectives during analysis. ATR is a senior qualitative researcher with extensive experience in qualitative and interpretive methodologies, contributing critical reflexivity and methodological rigor throughout the analytic process. MAS is a senior genetic counselor and educator with long‐standing experience in clinical genetics in the MENA region, providing supervisory and contextual insights. As researchers, we acknowledge that our cultural positions, professional roles, and disciplinary backgrounds actively shaped the analytic process, in line with RTA principles.

## 5 ANALYSIS

**FIGURE 1:**
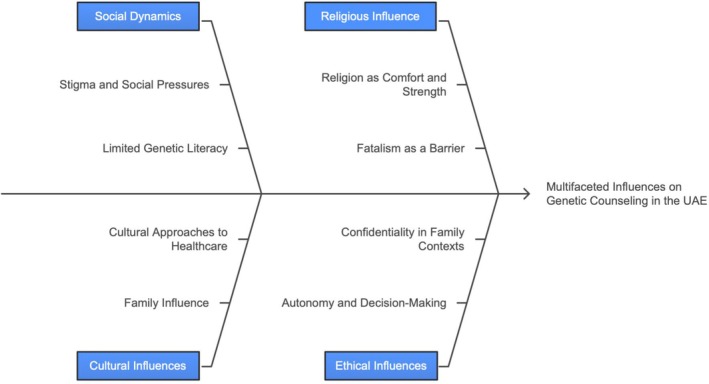
Themes and subthemes of barriers in genetic counseling in the UAE. The figure outlines four primary thematic categories: Social and cultural barriers, religious beliefs, and ethical challenges, each branching into detailed subthemes based on participant insights. The visualization highlights the complex interplay of sociocultural, religious, and ethical factors shaping service delivery.

Through our RTA, we constructed four main themes that illuminate how participants made sense of genetic counseling in the UAE. These themes represent patterns of shared meaning developed through our interpretive lens as researchers. The themes address social and cultural barriers, religious beliefs, ethical challenges, and systemic issues. Participants' narratives highlighted how stigma, varying levels of genetic literacy, and strong family influence shaped openness and decision‐making; how traditional beliefs complicated engagement; and how religious coping acted as both a facilitator and a deterrent. Participants described patients as often preferring directive guidance, with maintaining confidentiality made difficult by family involvement. Systemic gaps, including limited interprofessional coordination, insufficient time for informed consent, and lack of UAE‐specific training, were noted. In contrast, the Emirati Genome Program was considered a facilitator of awareness and access. Younger, educated patients were more proactive, highlighting the importance of targeted education and culturally sensitive, semi‐directive counseling approaches to improve genetic services as presented in the Ishikawa diagram (Figure 1).

### 5.1 Social dynamics of genetic counseling

Through participants' accounts, we constructed a theme highlighting the central role of social dynamics in shaping how families approached genetic counseling. Stigma, societal expectations, and limited awareness often created hesitation around disclosure and testing. Counselors noted that families sometimes minimized symptoms or avoided documentation, not out of resistance to healthcare, but as a way of protecting family image and managing sensitive information.

#### 5.1.1 Stigma and social pressures

Through our analysis, we constructed an interpretation of how social stigma operated as a powerful force shaping testing decisions. Concerns about social standing and marriage prospects influenced how openly families engaged with genetic counseling, and in some cases, discouraged them from pursuing genetic testing altogether.

Some families are very cautious about the stigma… and they would not proceed with genetic testing. (MGF03)

Others described how symptoms were downplayed to avoid attention or judgment.

Patients tend to downplay their children's symptoms because of stigma and cultural pressure. (GCF01)

When taking family history, people often say there's nothing significant, but later you find out there is. I think there's a tendency to just say everything is normal. (GCF02)

Beyond the clinic encounter, families expressed concern about information being shared across healthcare settings, which reinforced their caution in disclosure. Together, these accounts highlight how stigma shaped disclosure and decision‐making. Families sought to protect social reputation and future prospects, but these protective strategies also limited the accuracy of histories, complicated trust‐building, and constrained the uptake of services.

Beyond the clinic encounters, some people expressed fear that others might access their records, which reinforced their caution. (MGF03)

#### 5.1.2 Limited genetic literacy and its consequences

A further analytic thread focused on how limited familiarity with genetic concepts and terminology shaped patients' understanding of inheritance, risk, and testing. While this is not unique to the UAE, these gaps in knowledge complicated counseling, sometimes fostered blame within families, and made informed consent more difficult to achieve.

Counselors highlighted how numerical risk was often misinterpreted.

With autosomal recessive conditions, when we say there's a 25% risk, they think it means only the fourth pregnancy will be affected, or that if it happened once, it won't happen again. (MGF02)

Others described the misconception that genetic testing was a single, uniform procedure, rather than a range of options with different purposes and limitations.

A lot of people don't understand that genetic testing isn't just one thing. There are different types and layers. (GCF04)

Some patients also expressed beliefs that reflected partial or inaccurate understandings of inheritance.

Some patients think the father contributes the genes, and the mother is just a vessel. (GCM01)

Misattributions could extend to attributing traits or conditions to cultural explanations or pregnancy practices.

I had a patient with neurofibromatosis type 1 who thought her spots were because her mother didn't eat the food she craved during pregnancy. (GCF02)

A common misconception noted by participants was the tendency to over‐attribute conditions to consanguinity.

People tend to over‐attribute conditions to consanguinity. I've heard them say Down syndrome or autism happened because the parents are cousins, even though that's not how it works. (GCM01)

These examples show how gaps in genetic literacy were more than informational. They influenced how families understood causation, assigned blame, and weighed reproductive risks. Such misunderstandings underscored the importance of clear, culturally tailored education to support informed and autonomous decision‐making.

### 5.2 Cultural influences on engagement with genetic counseling

Through our analysis, we constructed a theme that highlights the central role of cultural norms and traditions in shaping engagement with genetic counseling services. This theme reflects how family dynamics, traditional belief systems, and culturally embedded approaches to illness shaped understanding of genetic risks and patterns of decision‐making. Our interpretation emphasizes that these cultural influences operated not as isolated factors, but as interconnected frameworks that shaped how patients and families navigated clinical encounters.

#### 5.2.1 Family influence

Our analysis illuminated the collective nature of healthcare decision‐making, where family is often central. Decision‐making was commonly positioned as a shared responsibility, reflecting cultural values of solidarity and familial cohesion. This collective model could be a source of emotional and practical support, but it also had the potential to shape or override individual preferences, requiring careful negotiation between personal agency and family expectations.

At the end, it was not one person's decision, but it was a family decision. (GCF02)

Other family members even help explain the disease and the management, because they had already been through it. (MGF03)

Our interpretation highlighted the crucial balance between respecting cultural context and protecting individual autonomy during clinical encounters.

It is crucial to respect patients' individuality and their right to determine their own course of decisions or their own course of life and try to kind of avoid the distraction from other family members. (GCM01)

#### 5.2.2 Cultural approaches to healthcare

Our analysis demonstrated the coexistence of traditional practices and cultural interpretations of illness with biomedical frameworks, which shaped families' interpretations of genetic conditions and their approach to healthcare decisions. Spiritual frameworks and traditional remedies were integrated into families' health‐seeking behaviors, illustrating the pluralistic ways in which illness was understood and managed.

The patient's mother told me there is a lady who came, and she cast an evil eye on him, and he became like this. I'm just going to take him to someone. They'll cast off this evil eye. (GCF04)

We have patients who use herbal medicine in metabolic conditions. (MGF04)

These accounts demonstrated that cultural beliefs were not necessarily in opposition to medical care but often shaped the timing and manner of engagement. Our interpretation emphasizes the importance of acknowledging and respectfully integrating these perspectives within counseling encounters, ensuring that culturally embedded beliefs are met with sensitivity while still providing accurate and actionable genetic information.

### 5.3 Religious influence

Our analysis highlighted the dual role of religion in shaping engagement with genetic counseling. On one hand, faith functioned as a source of comfort and resilience, supporting families as they processed difficult diagnoses and accepted outcomes as part of divine will. On the other hand, religious beliefs also influenced how some patients approached genetic testing or interventions, especially when they were perceived as conflicting with God's plan, requiring a delicate balance to ensure that respect for faith was balanced with the provision of accurate information and care.

#### 5.3.1 Religion as comfort and strength

Through our analysis, we constructed a theme that highlights how religious coping was narrated as a source of comfort and resilience. Trust in God (*tawakkul*) was described as enabling families to move from denial to acceptance, providing a spiritual framework for managing uncertainty and emotionally processing diagnoses.

This is a great support, and it's really supporting our patients to know that this is God's will… So, I think this is rather a positive thing rather than negative reinforcement. (GCF05)

Religion helps patients move from denial to acceptance. (GCF09)

Religious coping, however, was not monolithic; our analysis revealed that it could both reinforce engagement with services and at times create hesitations.

The concept of tawakkul, putting your trust in God, is like a two‐faced coin. It can enhance or impede the services. (GCM01)

#### 5.3.2 Fatalism as a barrier to care

A contrasting pattern constructed through our analysis was the role of fatalistic interpretations of faith in shaping healthcare decisions. In these accounts, religious belief was positioned as a potential barrier, with genetic testing or reproductive interventions sometimes framed as conflicting with divine will.

Some patients decline testing because they believe that whatever happens is God's will. (GCF06)

I've seen people who refuse abortion because of religious beliefs, although there is a genetic basis… even when we know the condition is lethal. (GCF04)

Our analysis also illuminated variation within these narratives: many families actively integrated faith and medical advice, while others adopted more fatalistic positions, illustrating a spectrum of interpretive approaches to religion and healthcare.

We have two categories of patients: some believe you need to leave everything to God, and others, who I'd say are the majority, accept our advice and work with it. (GCM01)

This theme highlights religion as a multifaceted force, simultaneously supporting coping and shaping decisions in ways that can both facilitate and limit engagement with genetic services. Counselors often balanced cultural sensitivity with the provision of accurate information and care.

### 5.4 Ethical influences in genetic counseling

Our analysis constructed a theme that highlights the ethical complexities shaping genetic counseling practice within the UAE context. These complexities reflected the interaction between professional standards and sociocultural expectations, particularly in relation to autonomy, confidentiality, and informed consent. The strong presence of family members in clinical encounters, alongside patients' tendencies to seek directive guidance, created ethically layered situations that required careful balancing of cultural sensitivity with professional principles.

#### 5.4.1 Autonomy and decision‐making

Through our analysis, we developed an interpretation of how autonomy was often experienced as relational rather than individual. Patients frequently positioned healthcare providers as the primary decision‐makers, expressing trust in clinicians as authoritative sources of knowledge. This dynamic, when combined with limited health literacy and strong family influence, shaped decision‐making processes within collective rather than individual frameworks.

I know that patients in many parts of the world, and the UAE is no exception, depend on doctors for making their own decisions. ‘You're the doctor, you know better, tell me what to do’. We encounter this very often in the clinic. (GCM01)

It's not just over here; all around the world, but a lot more over here in countries where health literacy is lower. Naturally, patients trust their doctors blindly. (GCF04)

Family input sometimes reinforced decisions, but in other instances, it redirected or constrained patient choice, reflecting how decision‐making unfolded within a collectivist and relational framework.

In some instances, family can have negative effects. Maybe discouraging a beneficial procedure… I've seen patients talk out of testing because the husband or other family members had a different agenda. (GCM01)

The Middle East, and especially the UAE, is very collectivistic. It's very different from Western society. There's a lot of input from multiple family members. (GCF06)

These accounts suggest that autonomy was often relational, negotiated between patients, families, and healthcare providers rather than exercised in isolation.

#### 5.4.2 Confidentiality in family‐centered contexts

Our analysis highlighted confidentiality as a particularly complex ethical terrain in collectivist settings, where families commonly attend appointments together. Within this context, maintaining patient privacy intersected with family dynamics and cultural expectations around shared decision‐making.

Sometimes patients come as a family, the spouse, the parents, and we try to draw the line, to respect the patient's decisions while also respecting the presence of family. (GCM01)

I've had situations where they wanted information about other family members, which I could not share. I had to deny those requests. (GCF02)

These accounts illustrate how confidentiality, while anchored in professional standards, was negotiated within a cultural framework that prioritizes collective engagement. Counselors often had to navigate these dynamics carefully, balancing ethical obligations with cultural sensitivity.

#### 5.4.3 Challenges in informed consent

The process of obtaining meaningful informed consent in genetic counseling was shaped by intersecting structural limitations and cultural dynamics. Limited consultation time, the complexity of genetic information, and the influence of family members often restrict the depth and quality of these discussions. Several accounts pointed to how current clinic structures do not allow sufficient time or space for thorough, individualized consent conversations.

Informed consent is really not given enough time. I believe it should be conducted in a separate clinic… At the moment, it's just not set up properly. (MGF01)

Sometimes people just say ‘I understand’ to sign and move on, but they don't really get the implications unless you take the time to explain in their own terms. (GCF04)

These narratives emphasize that informed consent is not merely a procedural formality but a relational and communicative process that requires adequate time, accessible explanations, and sensitivity to family involvement.

### 5.5 Institutional and contextual factors interpreted through the CFIR framework

Beyond social, cultural, religious, and ethical influences, our analysis illuminated broader institutional and systemic factors shaping the delivery of genetic counseling services in the UAE. Drawing on the CFIR as an interpretive lens, we deductively developed themes that reflected the interaction between inner and outer settings, as well as individual‐level characteristics, in shaping engagement with services (see Table 2).

**TABLE 2:** Constructed themes based on the consolidated framework for implementation research (CFIR).

Consolidated framework for implementation research (CFIR) domains			
Intervention characteristics	Characteristics of individuals	Inner setting	Outer setting
Knowledge is power Region‐specific training and professional development Promoting a culturally sensitive approach Supporting a semi‐directive counseling style	Sensitivities around specific conditions Age and proactivity	Coordination challenges between healthcare providers	Emirati genome program as a facilitator

#### 5.5.1 Emirati Genome Program as a facilitator (outer setting)

Our analysis revealed the Emirati Genome Program as a key external driver that supported greater public engagement with genetic services. Participants framed the program as an initiative that increased awareness, normalized discussions about genetics, and empowered patients to ask more informed questions during consultations.

Especially, I think with the Emirati Genome Program, the awareness of people starts to increase more and more about genetics… what it is exactly, and they are eager to hear from professionals. (MGF02)

People who've enrolled in the program come in and talk about it. It's clear they now have some background, they know a little more, they ask questions. It helped improve their genetic literacy. (GCM02)

These accounts illustrate how national initiatives can act as important facilitators for service uptake.

#### 5.5.2 Coordination between healthcare providers (inner setting)

We constructed a theme around coordination between genetic specialists and referring physicians as an institutional factor shaping service delivery. Our analysis revealed inconsistent referral practices and the circulation of oversimplified or inaccurate information, often linked to limited genetic literacy among nonspecialist providers. These dynamics placed additional demands on genetic counseling encounters, where counselors spent time correcting misunderstandings and resetting expectations.

Genetic literacy would be something that would enhance and fill the gap between the healthcare providers on one side and the genetic specialists on the other side. (GCM01)

You have physicians who refer to us, and they tell the patient it's just a blood test. Then we spend 30 min explaining why it's not that simple. (MGF01)

These examples underscore the importance of strengthening intra‐organizational networks and professional education to support more efficient service delivery.

#### 5.5.3 Sensitivities around specific conditions

##### Primary domain: Outer setting

###### Secondary domain: Characteristics of individuals

We constructed a theme focused on sensitivities attached to particular genetic conditions, including X‐linked disorders, neurodevelopmental conditions, and hereditary cancers. Our analysis positioned these sensitivities within broader cultural expectations and societal norms, which shaped how discussions about female carrier status, developmental delays, or genetic cancer risk were approached. While these sensitivities were experienced at the individual level, they were largely embedded in collective cultural frameworks, making the outer setting the primary domain. At the same time, emotional responses and knowledge gaps at the individual level aligned with the Characteristics of Individuals domain.

Some mothers seem unsure of how to respond to public reactions when their children have developmental delays. It can be a lot for them to manage. (GCF04)

There's sometimes a belief that males don't get breast cancer, or that only female relatives should be tested for BRCA‐related risks. We try to gently clarify those points. (GCM01)

This theme underscores how cultural norms intersect with individual understandings to produce complex communicative situations that require careful, culturally attuned responses.

#### 5.5.4 Age and proactivity (characteristics of individuals)

We interpreted generational differences as shaping patterns of engagement with genetic counseling. Younger and more educated patients were portrayed as more proactive, asking questions and making decisions informed by risk awareness and preventive thinking.

The younger generation asks more questions. They want to know, they want to prevent, and they don't just accept what the family says. (MGF03)

This interpretation points to knowledge, self‐efficacy, and readiness for change as important individual‐level factors influencing how patients engaged with counseling.

### 5.6 Practice‐related insights

**FIGURE 2:**
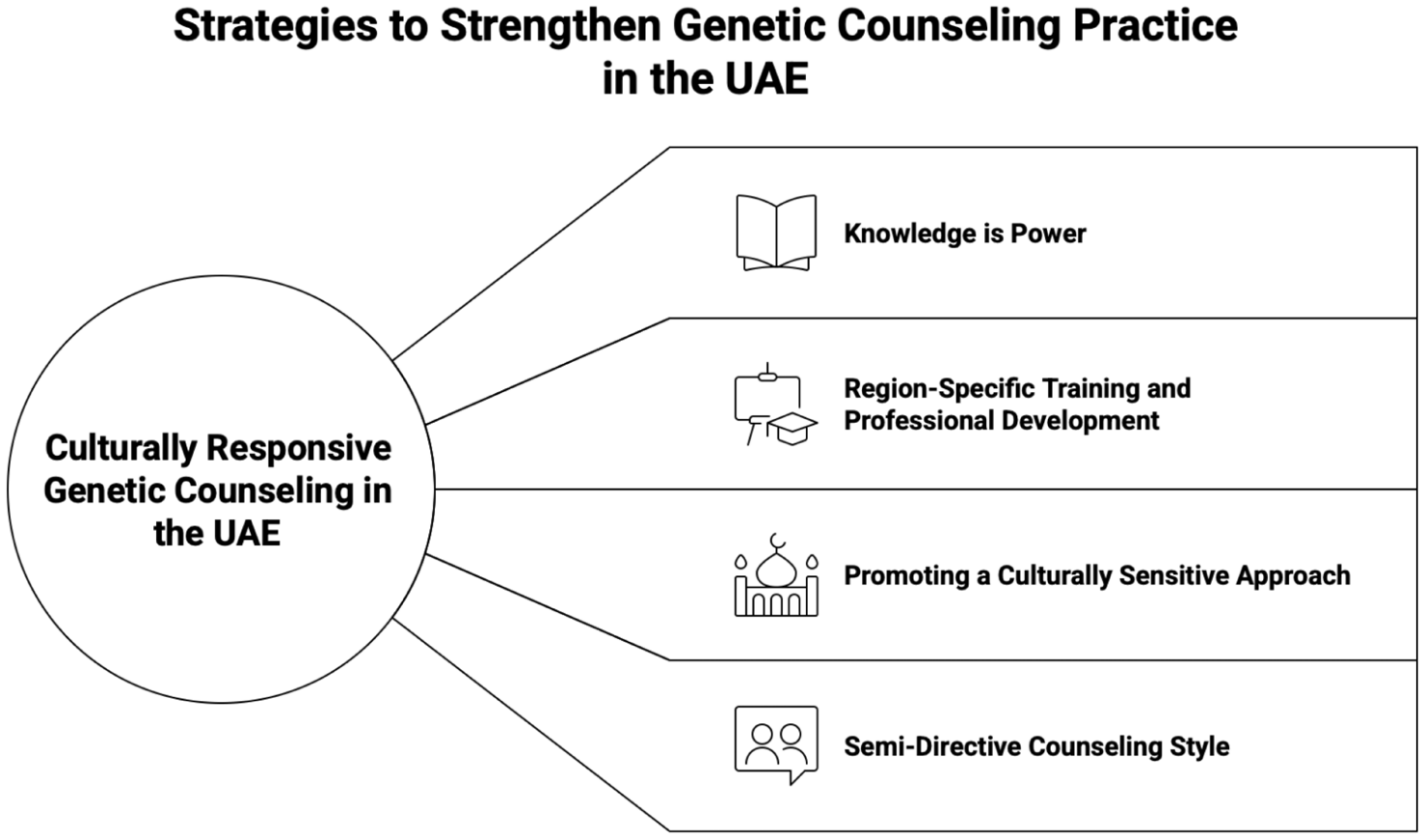
Strategies to strengthen the delivery of genetic counseling services. Visual summary of proposed strategies, including culturally adapted communication and UAE‐specific training.

Our analysis highlighted not only the barriers to genetic counseling but also the strategies articulated through participants' accounts to strengthen services. These strategies were grounded in their daily clinical experiences and emphasized the importance of approaches that balance cultural sensitivity with professional standards (see Figure 2).

#### 5.6.1 Knowledge is power

Our analysis underscored education as foundational to informed decision‐making. Education was framed as empowerment rather than persuasion, enabling patients to contextualize risk and options.

The more you empower someone with knowledge, the more they see it for themselves… You cannot dictate that this is how it has to be… Education is the key. (GCF04)

Our job is not to push anyone. We explain everything—the benefits, the risks, and the options, and help them see what's best for them and their families. (GCM01)

#### 5.6.2 Region‐specific training and professional development

We interpreted a clear need for professional development tailored to local cultural, familial, and religious dynamics, given that existing training often draws heavily on Western contexts.

In my master's program, we had some training about cultural considerations there, but not about the UAE. (GCF02)

Counseling here is very different; you really need to understand how family works, how people view testing, and what's acceptable or not. (GCF01)

#### 5.6.3 Promoting a culturally sensitive approach

Our analysis positioned cultural sensitivity as central to trust‐building and uptake. Models developed in Western contexts require adaptation to align with Emirati values and norms.

Our community has its own distinct religious and cultural values…we cannot simply follow Western models. (MGF01)

The government is supporting both the cultural value of consanguinity and the health of the future child. That balance is really commendable. (GCF04)

#### 5.6.4 Supporting a semi‐directive counseling style

We interpreted a semi‐directive counseling style as particularly well suited to the UAE context. This approach allowed counselors to provide clear, structured guidance while maintaining patient agency. In a setting where patients often seek explicit professional input and family involvement shapes decision‐making, semi‐directive counseling offered a balanced way to support autonomy while meeting cultural expectations.

I always try to follow a semi‐directive approach, give them the facts, guide them a bit, but leave the decision to them. Especially here, that works best. (GCF03)

We're not here to decide for them, but we do help them weigh things. It's not directive, but we do guide. (GCF01)

At the end of the day, we're just facilitators of decision. We give you the pros, cons, and help you think through it. Then, it's your choice. (GCM01)

## 6 DISCUSSION

This study constructed a comprehensive, interpretive overview of genetic counseling dynamics and the diverse barriers shaping its services in the UAE, representing the first interpretive account of its kind. Our analysis suggests that genetic counseling is shaped by overlapping social, cultural, religious, and ethical factors, all of which create unique obstacles for healthcare providers. While the global advancements of the field of genetic counseling are promising, the UAE context underscores the need for culturally sensitive services that align with local values and beliefs. A central theme we constructed from participants' narratives is social stigma. Our analysis highlighted how societal expectations and fear of repercussions shaped individuals' willingness to seek genetic services or disclose critical health information. In close‐knit communities, where social standing holds significant weight, genetic disorders were framed as threats to social standing. One counselor explained, “Some families are very cautious about the stigma… and they would not proceed with genetic testing.” (MGF03). This interpretation aligns with prior research from the Gulf region, which similarly highlighted the stigmatization of genetic disorders and its impact on healthcare access and engagement. (Al Aqeel, 2007; Jumah et al., 2019). Families with a history of genetic disorders were reported to face reduced marriage opportunities or rejection (El Shanti et al., 2015; Jassim et al., 2015). Stigma was not described in isolation but closely linked to broader systemic factors. Our analysis interpreted participants' accounts as pointing to the shortage of trained genetic counselors in our region exacerbating stigma's impact, leaving families with fewer resources to challenge or reframe negative perceptions (Jumah et al., 2019). Importantly, stigma is not unique to non‐Western societies. Findings from Portugal, where hereditary conditions prompted concealment across family, social, and institutional settings (Oliveira et al., 2025), reinforced that while stigma takes culturally specific forms, its impact on health care engagement is a cross‐cultural phenomenon. We interpreted stigma as compounded by low genetic literacy. Misconceptions about inheritance, recurrence risks, and gender‐based contributions to genetics were described as fueling avoidance of services and undermining trust. As one counselor reflected, “When taking family history, people often say there's nothing significant, but later you find out there is. I think there's a tendency to just say everything is normal.” (GCF02). Participants' narratives described cases where families over‐attributed conditions to consanguinity, misinterpreted recurrence risk as applying to the “fourth pregnancy,” or assumed that only fathers contributed genetic material. Such misunderstandings not only complicated communication but also fostered blame and hindered informed consent. This aligns with studies conducted in the UAE, reporting that only 7% of participants demonstrated adequate genetic literacy (Rahma et al., 2023), as well as international literature linking low literacy to disparities in care (Lea et al., 2011). In this way, stigma and limited literacy reinforced one another, jointly obstructing openness and informed engagement with genetic services. These misunderstandings were further entangled with traditional practices and explanatory frameworks. Our analysis interpreted participants' narratives as highlighting the reliance on supernatural explanations such as the evil eye or continued use of herbal medicine, which persisted alongside biomedical approaches. One counselor noted, “The patient's mother told me there is a lady who came, and she cast an evil eye on him, and he became like this. I'm just going to take him to someone. They'll cast off this evil eye.” (GCF04). Consanguineous marriages, while socially valued, were constructed as sensitive, as misconceptions about their risks often complicated counseling conversations. Patients were reported to attribute conditions such as Down syndrome or autism to cousin marriages, reflecting persistent gaps in understanding. Clarifying these risks was therefore considered requiring culturally sensitive counseling that respects traditions while offering accurate health information, consistent with Hamamy's (2012) work on consanguinity in Arab societies. At the same time, our analysis highlighted participants' framing of government initiatives as mediating these complexities structurally. Free genetic screening and IVF services (Department of Health – Abu Dhabi, 2023), alongside the expansion of the mandatory premarital screening program to over 580 conditions (Department of Health – Abu Dhabi, 2022), were described as mechanisms that uphold cultural values while minimizing genetic risks. These initiatives were widely commended by participants as innovative and culturally responsive policies. Also, religious beliefs also shape attitudes toward counseling in complex ways (Al‐Matary & Ali, 2014). Our analysis highlighted that for many families, faith was described as a buffer and a source of strength, with the concept of tawakkul (trust in God) supporting resilience and acceptance (Al Aqeel, 2007). These findings align with regional literature highlighting the protective role of religion in coping (Al Aqeel, 2007; Al‐Matary & Ali, 2014), as well as Western research documenting the psychological benefits of religious coping in contexts such as cancer care and bereavement (Braam et al., 2010; Loewenthal et al., 2001; McIntosh et al., 1993; Thune‐Boyle et al., 2013). However, religion was also interpreted as a potential source of constraint, particularly when framed fatalistically. Some families declined genetic testing or reproductive interventions, including termination of pregnancy, viewing them as interference with divine will, even when Islamic rulings would permit such actions under specific conditions. These dual interpretations highlight that religion cannot be framed as either purely a facilitator or a barrier, but functions as both, depending on interpretation and practice. Our analysis highlighted distinct ethical challenges, particularly concerning autonomy, confidentiality, and informed consent. Family involvement was consistently constructed as both supportive and constraining. While families offered solidarity and practical support, they could also exert pressures that sometimes overrode individual preferences. One counselor recalled, “In some instances, family can have negative effects. Maybe discouraging a beneficial procedure… I've seen patients talk out of testing because the husband or other family members had a different agenda.” (GCM01). This behavior reflects cultural norms of collectivism, where consensus is prioritized (Ezenkwele & Roodsari, 2013; Mobeireek et al., 2008). Confidentiality was difficult to maintain in contexts where patients attended sessions with multiple family members, blurring boundaries between privacy and collective responsibility. Patients were also described as deferring to providers, often expecting directive advice, consistent with hierarchical doctor–patient relationships in the region (Al‐Thani & Moore, 2012). While these dynamics challenge the Western ethos of nondirectiveness, they also create opportunities for ethical adaptation rooted in cultural responsiveness.

### 6.1 The semi‐directive model: A culturally aligned counseling approach

A central contribution of this study is the articulation of a semi‐directive model of genetic counseling, an approach specifically attuned to the sociocultural landscape of the UAE. In contrast to the Western emphasis on strict nondirectiveness, where counselors present information neutrally and refrain from influencing patients' decisions, participants described the limitations of neutrality in contexts of emotional distress, limited genetic literacy, or strong deference to healthcare providers.

We define semi‐directive counseling as a hybrid approach: one that upholds informed consent and respect for autonomy while allowing counselors to offer gentle guidance, clarify complex information, and recommend medically relevant next steps, particularly when patients are vulnerable or overwhelmed. This approach does not endorse paternalism. Instead, it is a form of context‐sensitive engagement that supports decision‐making.

This model resonates with existing frameworks that challenge rigid nondirectiveness. Biesecker & Peters et al. (2001) emphasized the genetic counselor's active role in facilitating patient understanding; Weil (2003) further critiqued the limitations of traditional nondirectiveness argued for a more relational model responsive to psychosocial context, and Elwyn et al. (2012) advanced the concept of shared decision‐making as a balance of guidance and autonomy. Together, these frameworks ground the semi‐directive model as a culturally responsive approach for settings where patients value relational input and ethical guidance. Participant narratives echoed these perspectives: “I always try to follow a semi‐directive approach—give them the facts, guide them a bit, but leave the decision to them. Especially here, that works best.” (GCF03); “We're not here to decide for them, but we do help them weigh things.” (GCF01). While most participants endorsed this, a few maintained a more traditional view of nondirectiveness, reflecting the evolution of professional norms and ongoing ethical debate.

While participants did not directly reference specific settings, this model may be relevant in scenarios such as when NIPT indicates a high risk for trisomy 18 or 13. In such situations, counselors might softly recommend confirmatory testing (e.g., CVS or amniocentesis) to ensure patients understand all reproductive options, including pregnancy termination within the UAE's legal window of 120 days gestation according to the Federal Law No. 4 of 2016 on Medical Liability (United Arab Emirates, 2016). Such guidance is not coercive but is ethically aligned with supporting timely, informed choices in a context where medical authority is highly valued. By contrast, strict nondirectiveness risks obscuring the urgency or significance of decisions, thereby undermining autonomy.

While the semi‐directive model presents a promising alternative to strictly nondirective counseling, its practical application raises essential ethical considerations, including the need for training in cultural competence, communication strategies tailored to varying literacy levels, and ongoing reflective supervision to avoid perceptions of paternalism.

**FIGURE 3:**
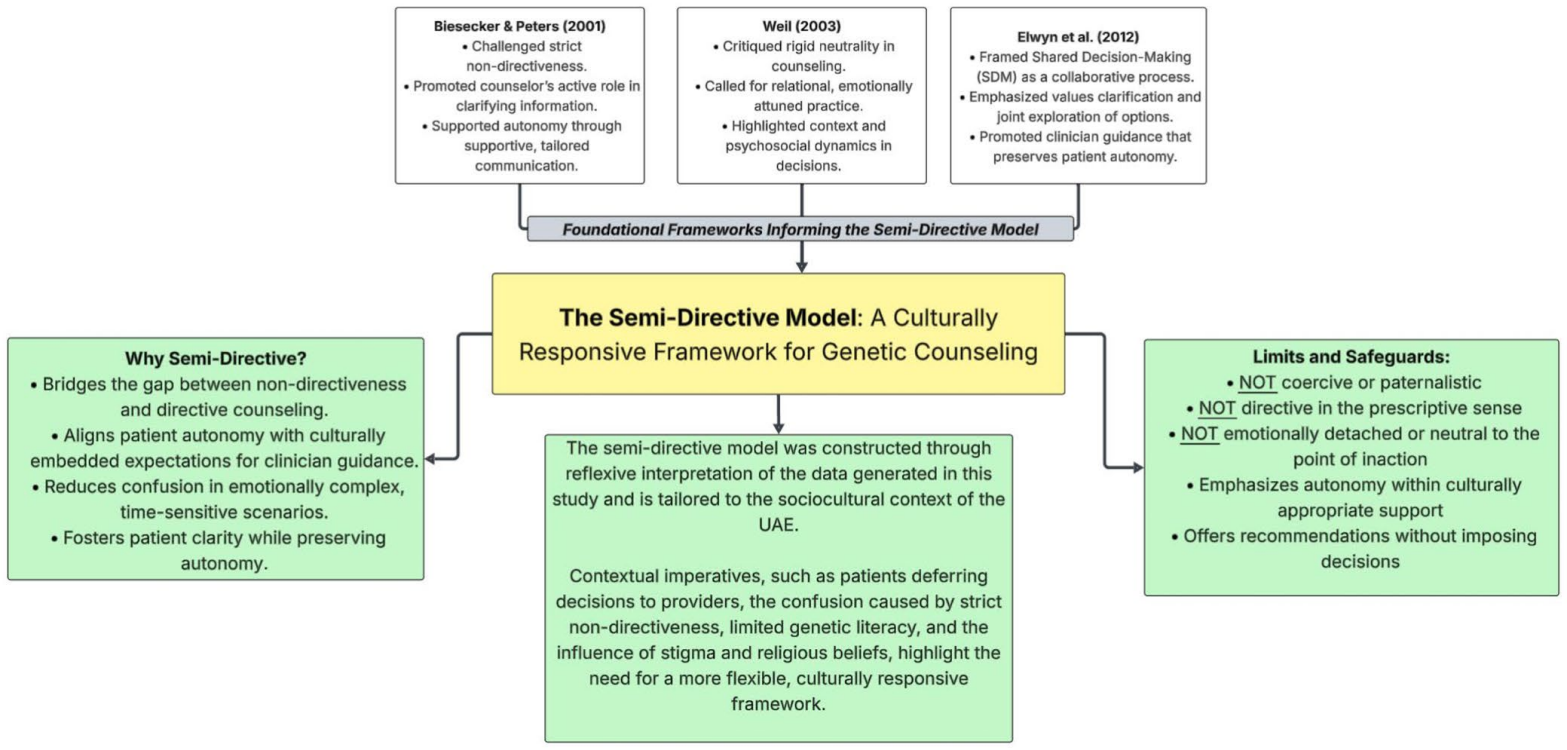
The semi‐directive model: a culturally responsive framework for genetic counseling. This figure synthesizes the conceptual foundations and empirical rationale underlying the semi‐directive model. It draws on prior frameworks that critique strict nondirectiveness, presents the inductive basis of the model as developed in this study, and outlines its intended use and ethical boundaries in the context of culturally embedded decision‐making.

We propose that the semi‐directive counseling approach merits further development as a framework for practice in contexts characterized by low health literacy, stigma, strong family involvement, and deference to authority. Although our analysis is grounded in the UAE, the model may also hold relevance for other collectivist societies where patients actively seek guidance from healthcare professionals. In this sense, our interpretation contributes not only to understanding practice in the UAE but also to wider debates about adapting genetic counseling frameworks across diverse cultural settings. Future research should assess its boundaries, training requirements, and adaptability across diverse cultural settings. These theoretical and empirical insights are visually synthesized in Figure 3, which illustrates how semi‐directiveness bridges neutrality and guidance, preserving autonomy while offering supportive input in contexts where relational engagement is expected.

In parallel with the development of the semi‐directive model, our analysis interpreted participants' narratives as highlighting a range of barriers and facilitators that shaped counseling experiences, including provider limitations, sociocultural pressures, and health system infrastructure. It interpreted participants' narratives as highlighting how policy‐related and institutional challenges intersected with the broader themes shaping genetic counseling practice. One notable challenge concerned the gap in genetic literacy among healthcare providers. Some accounts highlighted instances where patients received incomplete or inaccurate information from nonspecialists. Our analysis interpreted participants' narratives as highlighting gaps in the understanding and communication of X‐linked disorders, gendered perceptions of genetic conditions, and the stigma surrounding intellectual disabilities, which underscore the importance of tailored awareness campaigns. Similar challenges have been reported in previous studies from the MENA region and beyond (Clarke, 2016; Karam et al., 2024). The Emirati Genome Program was framed as an important facilitator in increasing public awareness, acceptance, and accessibility of genetic services and counseling. Taken together, these interpretations emphasize the need for multi‐level strategies to strengthen genetic counseling in the UAE. At the patient level, educational initiatives should demystify genetic concepts and normalize genetic conditions. At the provider level, UAE‐specific training and better coordination with nonspecialists are crucial to ensure accurate, culturally sensitive communication. At the system level, sustained national initiatives such as the Emirati Genome Program have proved to normalize discussions of genetics and enhance service uptake. The semi‐directive model developed through this analysis provides one culturally responsive framework to guide these efforts.

### 6.2 Strengths and limitations

A key strength of this study is its originality: it represents the first qualitative exploration of genetic counseling in the UAE, examining ethical, cultural, religious, and systemic influences. Drawing on in‐depth interviews with both genetic counselors and clinical geneticists provided diverse professional perspectives and rich, practice‐based knowledge. The use of RTA enabled the co‐construction of nuanced findings that would be difficult to capture through quantitative approaches. A notable conceptual contribution is the articulation of the semi‐directive model as a culturally adaptive framework, moving beyond the directive–nondirective dichotomy that dominates Western practice. Limitations include the relatively small sample size, which reflects the limited national workforce, and the exclusion of patients and family physicians in this phase, which may constrain the breadth of perspectives. The purposive sampling of professionals, while appropriate for the study aims, may limit transferability to other settings. These considerations should be kept in mind when interpreting the findings.

## 7 CONCLUSIONS

Through our interpretive analysis, we highlighted how cultural norms, religious beliefs, and family‐centered decision‐making shape genetic counseling practice in the UAE. Our findings demonstrate how stigma, low genetic literacy, religious fatalism, and ethical considerations around autonomy and confidentiality intersect to form key barriers. At the same time, we constructed an understanding of how national initiatives, such as the Emirati Genome Program, facilitate awareness and normalize genetic discourse. Drawing on participants' narratives, we proposed a culturally sensitive semi‐directive counseling model, supported by region‐specific training and system reforms to enable more patient‐centered care. As genomic medicine expands, our analysis underscores the need to address these context‐specific challenges to ensure services remain ethical, culturally congruent, and accessible. Future research should extend this work by incorporating patient perspectives and engaging policymakers to translate these insights into practice.

## AUTHOR CONTRIBUTIONS

**Hind J. Almarri:** Conceptualized the study, designed the interview guide, conducted the interviews, performed verbatim transcription, participated in thematic analysis and interpretation of results, wrote the first draft, and critically revised the manuscript. **Sameera Koodakkadavath:** Participated in thematic analysis and interpretation of results, wrote the first draft, and critically revised the manuscript. **Azhar T. Rahma:** Conceptualized the study, designed the interview guide, supervised data collection, participated in thematic analysis and interpretation of results, and critically revised the manuscript. **Muna Al Saffar:** Conceptualized the study, designed the interview guide, supervised data collection, and critically revised the manuscript.

## FUNDING INFORMATION

This research did not receive any specific grant from funding agencies in the public, commercial, or not‐for‐profit sectors.

## CONFLICT OF INTEREST STATEMENT

The authors declare no competing interests.

## ETHICS STATEMENT

Human studies and informed consent: This study adhered to the ethical principles outlined in the Declaration of Helsinki. Ethical approval was granted by the Social Science Ethical Review Committee at the United Arab Emirates University (Ref. ERSC_2023_3901, dated January 19, 2024). Informed written consent was obtained from all participants prior to the interview.

Animal Studies: Not applicable.

## Supporting information


File S1

## Data Availability

The transcripts generated and analyzed during the current study are available from the corresponding author upon reasonable request.
