# Supplementary material for: Voices in practice: Exploring genetic counseling ethical, cultural, social, and religious dynamics in the UAE
Source: J Genet Couns. 2025 Nov 14;34(6):e70139. doi: 10.1002/jgc4.70139 (PMC12619030; doi:10.1002/jgc4.70139)
Supplement: Supplementary file 1 — File S1 [file JGC4-34-0-s001.docx]

**Supplementary File – Interview Guide**

**Study Title**: *Voices in Practice: Exploring Genetic Counseling Ethical, Cultural, Social, and Religious Dynamics in the UAE*

*This semi-structured interview guide was used to explore how genetic counselors navigate sociocultural, ethical, and religious dimensions in their clinical practice within the UAE. The guide was designed to be flexible; not all questions were asked verbatim or in the same order. Follow-up probes were used to explore emerging themes in greater depth.*

**0. Demographic Information**

This brief section gathered background information to contextualize participants' responses and support analysis of professional diversity and experience.

Participants were asked to provide:

- Job title and role (e.g., genetic counselor, clinical geneticist)
- Years of experience in genetic counseling (total and within the UAE)
- Nationality and/or cultural background
- Languages spoken in clinical practice
- Type of institution (public/private, hospital/clinic)
- Emirate(s) of current practice
- Training background (e.g., country and type of genetic counseling training)

**1. Cultural Awareness and Training**

*This section explores whether counselors received training or preparation to support culturally competent practice within the UAE.*

- Have you received any specific training to prepare you for working within the cultural norms and expectations of the UAE?
- How has that training (or the lack of it) influenced your clinical approach?

**Probes:**

- Was the training formal or acquired through experience?
- Do you believe cultural competence should be a required component of training for genetic counselors practicing in the UAE?
- Have you encountered situations where a lack of cultural familiarity posed challenges?

**2. Traditional Beliefs and Practices**

*This section examines how counselors handle traditional beliefs, including folk explanations and healing practices that may arise during sessions.*

- How do you typically approach conversations involving traditional or folk beliefs during counseling sessions?
- Do patients often mention concepts like the “evil eye”? How do you respond in such cases?
- Can you describe a time when familiarity with traditional practices positively influenced a counseling encounter?

**Probes:**

- How do you balance respecting cultural beliefs with providing medically accurate information?
- Do you observe differences based on generational or regional backgrounds?

**3. Informed Consent and Autonomy**

*This section explores how counselors ensure informed consent while navigating family dynamics in a collectivist society.*

- How do you ensure that patients understand the implications of genetic testing and provide informed consent?
- In the context of our collectivist societies, to what extent are extended family members typically involved in the genetic counseling process, and how important is their role in shaping the patient's decision-making?
- How do you support the patient’s autonomy in decision-making, especially in cases of strong family involvement?

**Probes:**

- Have you faced situations where the patient appeared pressured by family?
- What strategies do you use to ensure the patient’s voice remains central?

**4. Social Stigma**

*This section explores the impact of social stigma on genetic counseling outcomes, including disclosure and testing decisions.*

- Based on your experience, how does social stigma surrounding genetic conditions influence the counseling process?
- What approaches have you found effective in helping patients navigate stigma?

**Probes:**

- Do patients express concern about disclosure within their community or family?
- How do you approach discussions about marriage and reproductive planning in stigmatized contexts?
- Are there particular genetic conditions that carry more stigma in your experience?

**5. Consanguinity**

*This section examines communication approaches when addressing consanguineous marriage and associated genetic risks.*

- Have you encountered patients who were unaware of the genetic implications of consanguineous marriage?
- How do you explain these risks in a way that is both informative and culturally sensitive?

**Probes:**

- How do you respond when patients minimize or deny the risks?
- Do you adjust your explanations based on education or background?

**6. Genetic Literacy**

*This section explores how counselors assess patients’ understanding of genetics and adapt their communication accordingly.*

- How do you assess a patient’s level of genetic knowledge before or during a session?
- How do you adjust your communication style based on their level of understanding?
- Have you encountered sessions where low genetic literacy significantly impacted the outcome?
- What misconceptions about genetics are commonly encountered, and how do you address them?
- How do you respond when patients bring misinformation from outside sources?
- Do you use visual aids or other tools to support patient understanding? If so, have you found certain methods to be more effective than others?

**Probes:**

- Do patients often confuse terms like “carrier” or “mutation”?
- In your experience, do factors such as a patient's education level, cultural background, or age influence their understanding of genetic concepts? If so, how do you adjust your approach accordingly?

**7. Religious Beliefs**

*This section focuses on how religious values influence decision-making and how counselors navigate faith-related concerns.*

- How do religious beliefs, such as the belief in God’s will, influence patients' acceptance or rejection of genetic testing or counseling recommendations?
- How do you approach religious practices or sensitivities during counseling sessions?

**Probes:**

- Have you seen religion influence decisions about prenatal testing or pregnancy continuation?
- Do you refer patients to religious authorities or fatwas when needed?
- Have you encountered challenges aligning clinical recommendations with religious beliefs?

**8. Non-directiveness**

*This section examines how counselors maintain non-directiveness and whether they incorporate personal disclosure.*

- How do you maintain a non-directive approach during sessions?
- What helps you avoid imposing personal values or opinions on patients?
- What are your views on using self-disclosure? Under what circumstances, if any, do you share personal experiences?

**Probes:**

- Have patients ever directly asked for your opinion or what you would do?
- Do you believe that maintaining a non-directive approach ever compromises trust with patients? If so, in what ways and how to avoid that?
- Can you give an example where self-disclosure was helpful or problematic?

**9. System-Level Reflections**

*This closing section explores broader reflections on integration, training needs, and system-level challenges.*

- In your opinion, how well is genetic counseling currently integrated into the healthcare system in the UAE, considering cultural, social, and religious dimensions?
- How do you stay informed about social and religious issues that may impact your practice?
- Do you think there is a need for additional training or support in this area?
- What approaches or strategies do you believe would help address the challenges related to social norms, religion, and culture in this field?

**Probes:**

- Do you feel supported by institutional guidelines or leadership when addressing these complexities?
- Have there been improvements in how genetic counseling services are delivered in culturally sensitive ways?
